# RNF126 writes a non-canonical ubiquitin code on midnolin to tune protein stability: RNF126 writes a non-canonical ubiquitin code on midnolin

**DOI:** 10.3724/abbs.2025232

**Published:** 2026-01-07

**Authors:** Yun Yang, Jin Ren, Xiang Qiu, Yanlin Liu, Shilin Yuan, Ronggui Hu, Zhixiong Xia, Chuanyin Li

**Affiliations:** 1 School of Pharmaceutical Science and Technology Hangzhou Institute for Advanced Study University of Chinese Academy of Sciences Hangzhou 310024 China; 2 State Key Laboratory of Molecular Biology Shanghai Institute of Biochemistry and Cell Biology Center for Excellence in Molecular Cell Science Chinese Academy of Sciences Shanghai 200031 China; 3 Department of Colorectal Surgery and Oncology (Key Laboratory of Cancer Prevention and Intervention China National Ministry of Education Key Laboratory of Molecular Biology in Medical Sciences Zhejiang Province China) the Second Affiliated Hospital Zhejiang University School of Medicine Hangzhou 310009 China; 4 Center for Medical Research and Innovation in Digestive System Tumors Ministry of Education Hangzhou 310009 China

**Keywords:** MIDN, RNF126, non-lysine ubiquitination, proteasomal degradation, EGR1

## Abstract

Midnolin (MIDN) is a newly recognized master regulator that drives ubiquitin-independent proteasomal degradation, yet the mechanisms governing its own turnover remain enigmatic. Here, we demonstrate that MIDN is ubiquitinated and identify RNF126 as the cognate E3 ligase. RNF126 physically associates with MIDN and catalyzes its ubiquitination, and mass spectrometry mapping reveals that this process occurs primarily at non-canonical cysteine, serine, and threonine residues (C230, C236, S237, T239, and S241) rather than at lysine residues. This non-classical ubiquitination targets MIDN for 26S-proteasomal degradation. *In vivo* dissection of the RNF126-MIDN axis shows that it governs EGR1 abundance and, consequently, the tumor-suppressor proteins PTEN and p53, thereby restraining the progression of testicular germ-cell tumors (TGCTs). Our findings reveal an unappreciated layer of MIDN regulation and identify the RNF126-MIDN ubiquitination cascade as a potential therapeutic vulnerability in TGCTs and related malignancies.

## Introduction

Protein homeostasis underpins every cellular decision-whether division, differentiation, stress adaptation, or death-by maintaining the proteome in precise balance across its abundance, quality, spatial distribution, and temporal dynamics [ 1– 5]. Its dysregulation underpins a spectrum of human diseases, including cancers and diverse neurological disorders [ 6 – 12]. The ubiquitin-proteasome system (UPS) is the principal engine of proteostasis in eukaryotes. By covalently attaching the 76-amino-acid protein ubiquitin to internal lysine residues of substrate proteins, E3 ubiquitin ligases generate a combinatorial code (mono-, multi or polyubiquitin chains of varying linkage topology) that is decoded by proteasomal receptors, de-ubiquitinating enzymes and other effectors [ 2, 13]. Canonical K48-linked polyubiquitin chains are the best-characterized signal for 26S proteasomal degradation, but noncanonical linkages (K11, K63, M1, *etc*.) and non-lysine acceptor sites are increasingly recognized as vital layers of regulatory complexity [ 2, 14, 15].

Midnolin (MIDN) has recently emerged as a fascinating nexus between transcriptional regulation and protein stability. Initially, annotated as a nucleolar protein, MIDN unexpectedly drives the 26S proteasomal degradation of a select cohort of transcription factors ( *e*. *g*., EGR1, FosB, and NR4A1) in a ubiquitination-independent manner [ 16, 17]. Through this ubiquitination-independent proteasomal activity, MIDN governs distinct developmental and pathological programs-neuronal lineage progression, T-cell immune responses, and oncogenic signaling by selectively targeting discrete substrate transcription factors in each context [ 18 – 20].

Given the inherently short half-life of MIDN, its intracellular abundance must be stringently controlled; however, the molecular machinery underpinning this regulation remains to be elucidated. Whether MIDN is subject to ubiquitination and whether such post-translational modification modulates its biological function are unresolved. Here, we establish RNF126 as the E3 ubiquitin ligase for MIDN and systematically investigate how subsequent ubiquitination controls MIDN proteostasis and downstream signaling.

## Materials and Methods

### Plasmid construction

Plasmids encoding MIDN and RNF126 were constructed by amplifying full-length or truncated cDNA from HEK293T cells and cloning the products into PACYC, pcDNA3.0-Flag/MYC, pET22b, pCDH or pGEX-4T-1. Point mutations were introduced by site-directed mutagenesis as previously described [21].

Single-guide RNAs (sgRNAs) targeting RNF126 and MIDN were designed, synthesized as oligos (YOCON Biotechnology, Beijing, China), annealed, and ligated into *Bsm*BI-digested lentiCRISPR v2 (Thermo Fisher Scientific, Waltham, USA) with T4 DNA ligase (NEB, Boston, USA). The sequences are listed in Supplementary Table S1.

### Cell culture, transfection and reagents

Human embryonic kidney 293T (HEK293T) cells were obtained from the Cell Bank of the Chinese Academy of Sciences (Shanghai, China) and maintained in high-glucose DMEM (Witcel, Shanghai, China). The human seminoma line TCam-2 was purchased from the American Type Culture Collection (Manassas, USA) and cultured in RPMI-1640 (Witcel). Both media were supplemented with 10% fetal bovine serum (FBS; Gibco, Carlsbad, USA), 100 U/mL penicillin and 100 μg/mL streptomycin (Gibco). The cells were maintained at 37°C in a humidified incubator with 5% CO _2_. The cells were transfected with plasmids using T103 LipoMAX 3000 transfection reagent (Witcel) following the manufacturer’s protocol. Stable cell lines were generated by continuous selection with 2 μg/mL puromycin (Beyotime, Shanghai, China) for at least seven days. Cycloheximide (CHX, Selleck, USA) was prepared as a 100 mM stock in dimethyl sulfoxide (DMSO) and stored at –80°C.

### Yeast two-hybrid screen

Yeast two-hybrid screening was carried out as previously described [22], with full-length human MIDN cloned as bait. Positive colonies were first selected on SD-4 (deficient in Ura, His, Leu and Trp) media and then assayed for β-galactosidase activity by X-Gal staining (Sangon Biotech, Shanghai, China). Plasmids from verified positives were rescued and sequenced to identify the corresponding prey fragments.

### Recombinant protein purification

GST- and His6-tagged proteins were expressed in *E*. *coli* BL21 (DE3). Expression was induced with 1 mM IPTG (isopropyl β-D-1-thiogalactopyranoside; Sangon Biotech) for 12 h at 37°C. The cells were harvested by centrifugation, resuspended in PBS (pH 8.0) containing 1 mM PMSF (Beyotime), and lysed by sonication. Clarified lysates were incubated with glutathione-Sepharose 4B or Ni-NTA agarose (Biolinkedin, Shanghai, China) for 4 h at 4°C. The beads were washed with PBS containing 0.1% Triton X-100, and the proteins were eluted with 20 mM reduced L-glutathione (pH 8.0) or 500 mM imidazole (pH 8.0). The eluates were dialyzed overnight at 4°C against PBS containing 20% glycerol, snap-frozen in liquid nitrogen, and stored at –80°C.

### GST pull-down assay

Purified His6-tagged protein (5 μg) and GST-tagged protein (5 μg) were incubated overnight at 4°C with 50 μL of glutathione Sepharose 4B in 1 mL of ice-cold GST pull-down buffer (20 mM Tris-HCl pH 8.0, 100 mM NaCl, 5 mM MgCl _2_, 1 mM EDTA, 0.5% NP-40, 1 mM DTT, and 10 mg/mL fresh BSA). The resin was collected by brief centrifugation and washed three times with 1 mL of the same buffer. The bound proteins were eluted by boiling in 50 μL of 2× SDS loading buffer for 10 min and analyzed by immunoblotting.

### Fluorescence microscopy analysis

HEK293T cells were transfected for 24 h with plasmids encoding RNF126-mCherry and GFP-MIDN, fixed with 4% paraformaldehyde (Beyotime), and counterstained with DAPI (Beyotime). Images were acquired on a BX51 fluorescence microscope (Olympus, Tokyo, Japan).

### Co-immunoprecipitation (Co-IP), immunoprecipitation (IP) and immunoblotting (IB)

For the Co-IP assay, HEK293T cells were lysed in 1 mL of Co-IP buffer (50 mM Tris-HCl, 150 mM NaCl, 5 mM EDTA, and 1% NP-40, pH 7.6) supplemented with a protease-inhibitor cocktail (Selleck, Houston, China). After centrifugation, the supernatant was incubated overnight at 4°C with an anti-RNF126 antibody (1:100 dilution, 66647-1-Ig; Proteintech, Wuhan, China) or control mouse IgG (1:100 dilution, B900620; Proteintech) and protein G magnetic beads (L-1002; Biolinkedin). For the IP assay, cells transfected with the indicated plasmids were lysed in 500 μL of RIPA buffer (150 mM NaCl, 50 mM Tris-HCl, 5 mM EDTA, 1% NP-40, and 0.1% SDS, pH 8.0) freshly supplemented with protease-inhibitor cocktail. The lysates were incubated overnight at 4°C with anti-Flag affinity gels. The next day, the immunoprecipitates were washed three times with Co-IP/IP buffer and denatured at 100°C for 10 min in 50 μL of 2× SDS loading buffer. Immunoprecipitates and input samples were resolved on 10% or 15% SDS-PAGE gels and transferred to PVDF membranes (Bio-Rad, Hercules, USA). The membranes were blocked with 10% skim milk for 1 h at room temperature and then incubated overnight at 4°C with the following primary antibodies (all from Proteintech): anti-Flag (1:2000; 80010-1-RR), anti-GAPDH (1:5000; 60004-1-Ig), anti-ubiquitin (1:2000; 10201-2-AP), anti-GST-HRP (1:10,000; HRP-66001), anti-His-HRP (1:1000; HRP-66005), anti-RNF126 (1:1000), anti-MIDN (1:1000; 18939-1-AP), anti-EGR1 (1:1000; 55117-1-AP), anti-p53 (1:1500; 10442-1-AP) or anti-PTEN (1:1000; 60300-3-Ig). After washing, the membranes were incubated for 1 h at room temperature with the following HRP-conjugated secondary antibodies (Proteintech): goat anti-rabbit IgG (1:5000; SA00001-2) or goat anti-mouse IgG (1:5000; SA00001-1). The signals were visualized with a Tanon 5200 imaging system (Tanon, Beijing, China).

### Reconstitution of the *E*. *coli* ubiquitination system and mapping of MIDN ubiquitination sites

E1 (UBA1), E2 (UBCH5A), and HA-Ub were inserted into the first multiple cloning site (MCS) of pACYC-Duet, whereas RNF126 was inserted into the second MCS to generate pACYC-HA-Ub-UBCH5A-UBA1-RNF126. Similarly, the control plasmid pACYC-HA-Ub-UBCH5A-UBA1 (lacking RNF126) was constructed. Each pACYC derivative was co-transformed with pET22b-MIDN-His6 into BL21 (DE3) competent cells, and transformants were selected on LB agar supplemented with ampicillin (100 μg/mL; Sangon Biotech) and chloramphenicol (50 μg/mL; Sangon Biotech). After induction with 2.5 mM IPTG overnight at 16°C, the cells were lysed in 8 M urea buffer (50 mM Tris-HCl, 50 mM Na _2_ HPO _4_, 300 mM NaCl, 8 M urea, 0.5% NP-40, 20 mM imidazole, pH 8.0) and rotated with Ni-NTA agarose for 4 h at room temperature. The resin was washed three times with the same urea buffer, and the bound proteins were eluted by heating in 2× SDS loading buffer at 100°C for 10 min prior to immunoblotting.

Ubiquitinated MIDN recovered from the *E*. *coli* system was processed for ubiquitin site mapping as previously described [23]. The protein was dissolved in 8 M urea and 100 mM Tris-HCl (pH 8.0), reduced with TCEP, alkylated with NEM, and digested with trypsin. Peptides were separated on an EASY-nLC system (Thermo Fisher Scientific) and analyzed on a Q Exactive mass spectrometer (Thermo Fisher Scientific). The data were processed with Proteome Discoverer 2.1 (Thermo Fisher Scientific) against the UniProt Human database ( www.uniprot.org). Detailed information on the ubiquitination sites of MIDN is provided in Supplementary Table S2, and the mass spectrometry data have been deposited in the ProteomeXchange Consortium via the PRIDE partner repository under the dataset identifier PXD069387.

### Generation of knockout cell lines

*RNF126*- and *MIDN*-knockout (KO) cells were generated by CRISPR-Cas9 as previously described [21]. HEK293T or TCam-2 cells were transfected with lentiCRISPR v2 carrying gene-specific sgRNAs and selected with 2 μg/mL puromycin for 2 weeks; complete loss of protein was verified by immunoblotting.

### Structure prediction of the MIDN-RNF126 complex

Structure prediction of the MIDN-RNF126 protein complex was performed using AlphaFold Server ( https://alphafoldserver.com/). The highest-ranked model was selected for downstream analysis based on AlphaFold3 confidence scores (ipTM+ptm). Protein–protein interaction interfaces were visualized using PyMOL, and interfacial residues within a distance of < 5 Å from the binding partner were identified. Subsequently, key residues potentially engaged in non-covalent interactions-including hydrogen bonds, salt bridges, and π-π stacking-were identified based on structural features and annotated with their residue numbers.

### Tumor xenograft assay

Four-week-old athymic male nude mice were purchased from Shanghai SLAC Laboratory Animal Co., Ltd. (Shanghai, China) and acclimated for one week before tumor cell injection. All animal procedures were conducted in accordance with the guidelines approved by the Institutional Animal Care and Use Committee of the Shanghai Institutes for Biological Sciences, Chinese Academy of Sciences (approval No. 2022-135). Tumor cells (TCam-2) of the indicated genotype were subcutaneously injected into the mice (1 × 10 ^7^ cells per mouse, six mice per group). Owing to the slow tumor growth of this cell line, tumor volumes were measured every 10 days using a vernier caliper, and calculated using the following formula: volume (V) = 1/2 × (length × width ^2^). On day 50, the mice were sacrificed, and the tumor weights were recorded. Tumors were then lysed in RIPA buffer and subjected to immunoblotting analysis.

### Statistical analysis

All the data are presented as the mean ± standard deviation (SD) and were analyzed using GraphPad Prism 7.0 (GraphPad Software, San Diego, USA). Comparisons between two groups were performed with a two-tailed unpaired Student’s *t* test, whereas multi-group comparisons were conducted by one-way ANOVA followed by Tukey’s *post hoc* test. A two-tailed *P* < 0.05 was considered statistically significant.

## Results

### RNF126 physically interacts with MIDN

**Figure 1:**
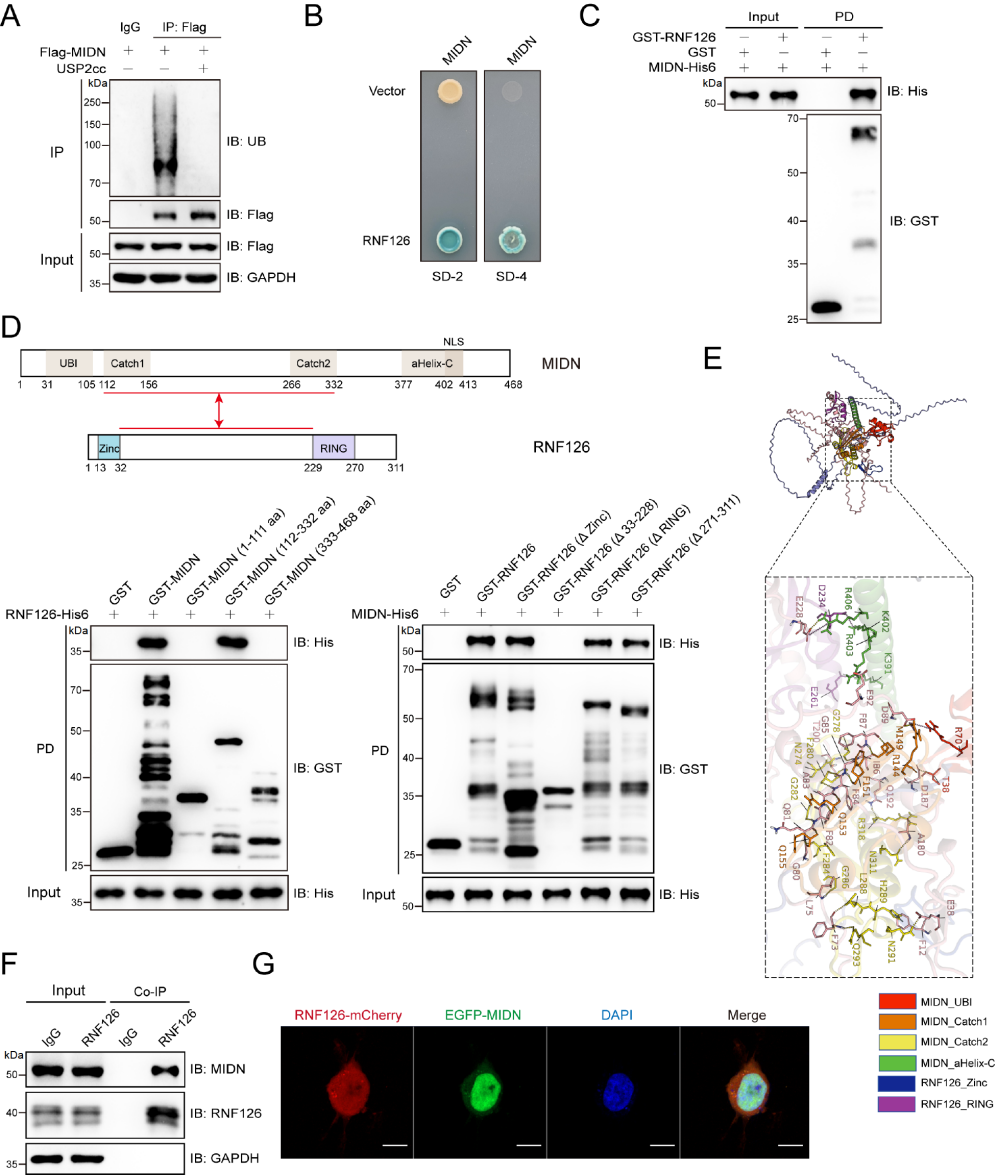
Figure 1 RNF126 is identified as an interactor of MIDN (A) HEK293T cells expressing Flag-MIDN were lysed, immunoprecipitated with anti-Flag (or control IgG), incubated with or without recombinant USP2 cc (USP2 catalytic domain), and immunoblotted as indicated.(B) Yeast two-hybrid (Y2H) screening identified RNF126 as an interacting partner for MIDN. MIDN was used as bait, SD-2, which is deficient in Leu and Trp, and SD-4, which is deficient in Ura, His, Leu and Trp. (C) Recombinant RNF126 directly interacted with MIDN. A GST pull-down assay was performed with recombinant GST-RNF126 and MIDN-His6. (D) The interaction between MIDN and RNF126 was localized to residues 112-332 of MIDN and 32-229 of RNF126. The indicated recombinant GST- or His6-tagged proteins were purified and used in GST pull-down assays. (E) Structural model of the RNF126-MIDN interaction predicted by AlphaFold 3 and rendered in PyMOL. (F) Endogenous MIDN associates with the E3 ubiquitin ligase RNF126. MIDN was immunoprecipitated from HEK293T cell lysates with an anti-MIDN antibody (or control IgG) and immunoblotted as indicated. (G) Exogenously expressed RNF126 and MIDN colocalized in the nucleus of HEK293T cells. Plasmids encoding RNF126-mCherry and EGFP-MIDN were co-transfected into HEK293T cells, and their fluorescence was visualized by microscopy. Scale bar: 10 μm.

To explore MIDN homeostasis, exogenous Flag-tagged MIDN was expressed in HEK293T cells, and ubiquitination was detected ( Figure 1A), indicating that MIDN is also regulated by the ubiquitin–proteasome system (UPS). To identify the E3 ubiquitin ligase responsible for MIDN ubiquitination, a yeast two-hybrid (Y2H) screen was performed, and RNF126 was identified as a candidate E3 ligase ( Figure 1B), with the interaction further validated by a GST pull-down assay ( Figure 1C). Deletion mapping narrowed the binding surface to residues 112--332 of MIDN and 32--229 of RNF126 ( Figure 1D), a topology consistent with the AlphaFold3-predicted complex ( Figure 1E). Endogenous MIDN was found to complex with the E3 ubiquitin ligase RNF126 ( Figure 1F). Fluorescence microscopy further revealed that mCherry-RNF126 and GFP-MIDN co-localize predominantly in the nuclei of HEK293T cells ( Figure 1G).

### RNF126 ubiquitinates MIDN on non-Lys residues

**Figure 2:**
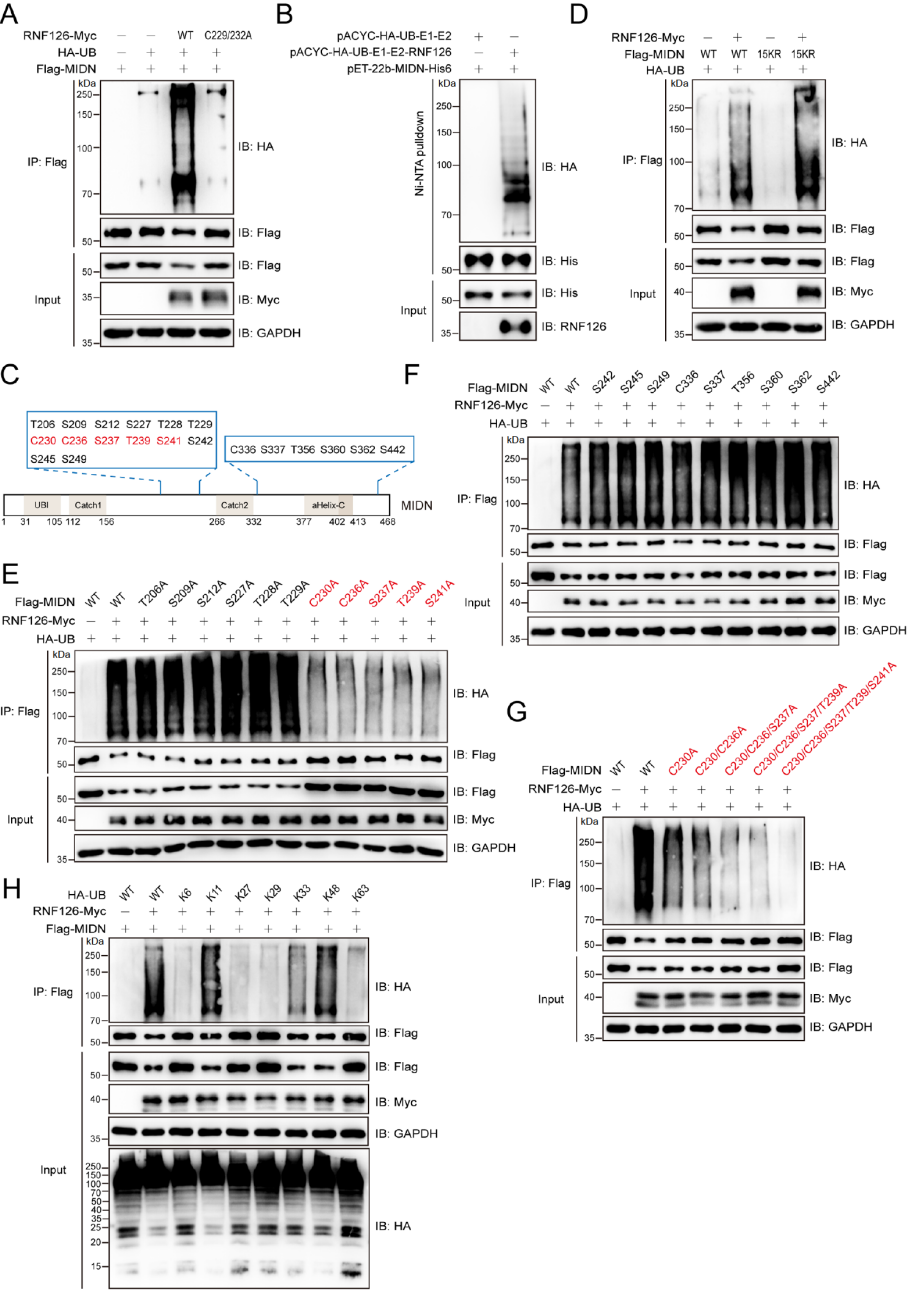
Figure 2 RNF126 ubiquitinates MIDN on non-Lys residues (A) Flag-MIDN was efficiently ubiquitinated by Myc-RNF126. HEK293T cells were co-transfected with the indicated plasmids; lysates were immunoprecipitated with anti-Flag affinity gel and analyzed by immunoblotting. RNF126-C229/232A is an E3-ligase-dead mutant. (B) RNF126 ubiquitinated MIDN in a reconstituted E. coli ubiquitination system. pACYC-E1(UBA1)-E2(UBCH5A)-RNF126/PACYC-E1(UBA1)-E2(UBCH5A) and pET22B-MIDN were co-transformed into BL21 E. coli cells, and protein expression was induced by IPTG. His6-MIDN was affinity purified on Ni-NTA resin, and the resulting ubiquitin adducts were visualized by immunoblotting. (C) Schematic distribution of the twenty sites involved in the RNF126-mediated ubiquitination of MIDN. The MIDN protein was recovered from the E. coli ubiquitination system (B) and subjected to mass spectrometry analysis to identify the ubiquitination sites. (D) Ubiquitination of MIDN by RNF126 persists after all lysines (K) are mutated to arginines (R), indicating lysine-independent modification. HEK293T cells were co-transfected with the indicated plasmids; lysates were immunoprecipitated with anti-Flag affinity gel and analyzed by immunoblotting. (E–G) RNF126-mediated ubiquitination of MIDN occurs primarily at five major sites: C230, C236, S237, T239 and S241. Lysates from HEK293T cells ectopically expressing the indicated plasmids were immunoprecipitated with anti-Flag affinity beads and analyzed by immunoblotting. (H) RNF126 catalyzes MIDN ubiquitination predominantly via K11- and K48-linked chains. HEK293T cells were co-transfected with RNF126-Myc, Flag-MIDN, and HA-Ub (either WT or the single-lysine mutants K6, K11, K27, K29, K33, K48, or K63, in which only the indicated lysine residue is intact while all others are mutated to arginine). The cell lysates were immunoprecipitated with anti-Flag affinity gel and analyzed by immunoblotting with the indicated antibodies.

After RNF126 was established as an interacting partner of MIDN, ubiquitination assays were subsequently performed. Exogenously expressed MIDN was efficiently ubiquitinated by RNF126 but not by its catalytically inactive C229/232A mutant ( Figure 2A). This ubiquitination was recapitulated in a fully reconstituted *E*. *coli* ubiquitination system lacking endogenous E3s, confirming that RNF126 is sufficient to ubiquitinate MIDN ( Figure 2B). The MIDN proteins recovered from the *E*. *coli* ubiquitination system ( Figure 2B) were analyzed by mass spectrometry, which identified ubiquitination at twenty non-lysine residues—specifically, cysteine, serine, and threonine residues ( Figure 2C). Mutation of all 15 lysine residues to arginine residues in MIDN (15KR mutant) did not diminish RNF126-mediated ubiquitination, whereas combined alanine substitution of the five most frequently modified residues, C230, C236, S237, T239 and S241 (5A mutant), almost completely abolished ubiquitination signaling ( Figure 2D–G). Ubiquitin linkage analysis revealed that RNF126 preferentially assembles K11- and K48-linked polyubiquitin chains on MIDN via HA-Ub mutants, in which only the indicated lysine (K11 or K48) is intact and all others are mutated to arginines ( Figure 2 H).

### RNF126 triggers 26S proteasome-dependent turnover of MIDN

**Figure 3:**
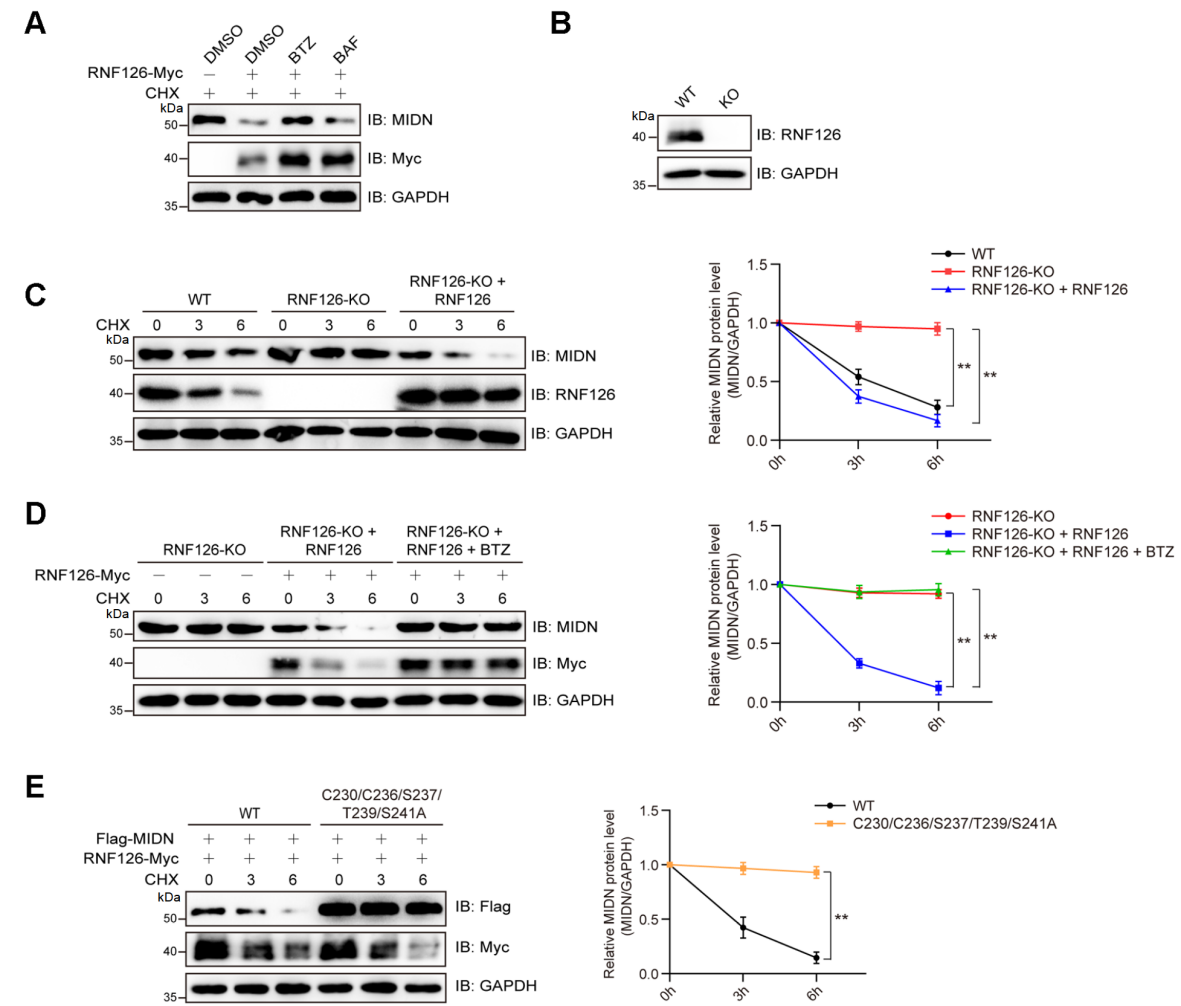
Figure 3 RNF126 mediates MIDN degradation (A) RNF126 mediates MIDN degradation primarily via the proteasomal pathway. HEK293T cells were treated for 6 h with bortezomib (BTZ, 1 μM), bafilomycin A1 (BAF, 20 nM), or cycloheximide (CHX, 100 μg/mL) before harvest. (B) Validation of RNF126 knockout efficiency. HEK293T cells were transfected with CRISPR-Cas9-sgRNA plasmids targeting RNF126 and selected with puromycin. (C) RNF126 depletion stabilizes MIDN, and this effect is reversed upon reintroduction of RNF126. HEK293T cells, with or without RNF126 transfection, were treated with CHX for the indicated durations and then subjected to immunoblotting analysis. The experiment was repeated three times, and the data were subjected to statistical analysis. **P < 0.01. (D) RNF126-mediated degradation of MIDN was blocked by the proteasome inhibitor bortezomib (BTZ). RNF126-knockout HEK293T cells, with or without reintroduction of RNF126, were treated with CHX for the indicated times and analyzed by immunoblotting. The experiment was repeated three times, and the data were subjected to statistical analysis. **P < 0.01. (E) RNF126 destabilized wild-type MIDN but not the 5A mutant (C230A/C236A/S237A/T239A/S241A). HEK293T cells were co-transfected with RNF126-Myc and either Flag-MIDN or the 5A mutant, treated with cycloheximide for the indicated times, and analyzed by immunoblotting. The experiment was repeated three times, and the data were subjected to statistical analysis. **P < 0.01.

In HEK293T cells, the RNF126-dependent decrease in MIDN abundance was completely attenuated by the proteasome inhibitor bortezomib (BTZ) but remained unaffected by the autophagy inhibitor bafilomycin A1 (BAF) ( Figure 3A). CRISPR/Cas9-mediated *RNF126*-KO cells were established ( Figure 3B). Loss of RNF126 blocked MIDN degradation, whereas re-introducing wild-type RNF126 restored its rapid turnover ( Figure 3C). Bortezomib (BTZ) treatment stabilized MIDN in *RNF126*-knockout cells even after re-expression of RNF126 ( Figure 3D). Importantly, the ubiquitination-deficient 5A mutant of MIDN resisted RNF126-mediated degradation ( Figure 3E), demonstrating that these non-canonical ubiquitin signals are essential for MIDN turnover.

### The RNF126-MIDN axis governs EGR1 signaling

**Figure 4:**
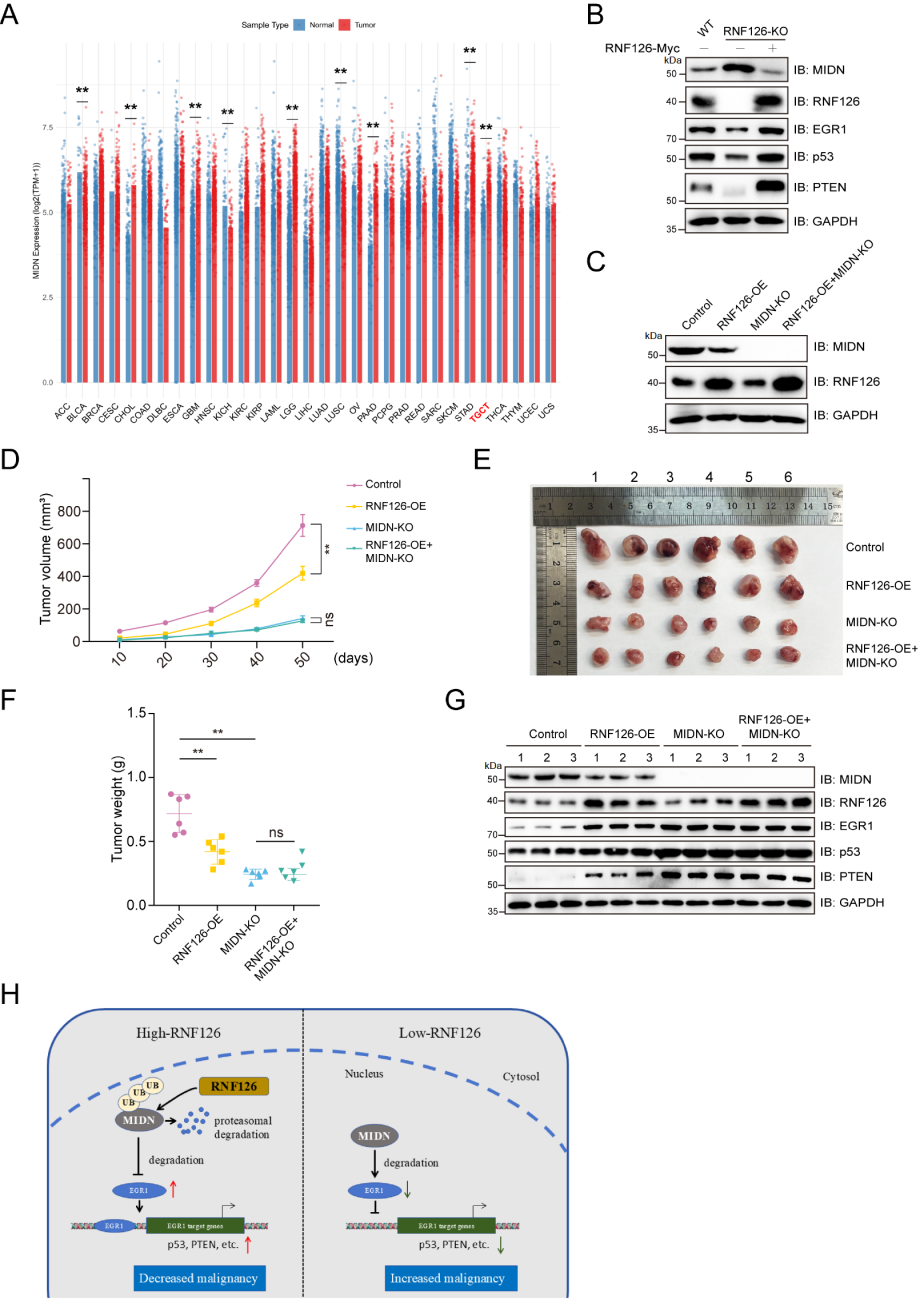
Figure 4 RNF126 regulates the downstream signaling pathways of MIDN-mediated, ubiquitin-independent degradation (A) Analysis of MIDN expression across different tumor types. The expression data containing TCGA and GTEx samples were downloaded from the UCSC Xena database, with GTEx samples serving as supplementary normal controls for various cancer types. R’s ggplot2 package was used to generate bar plots, and the Wilcoxon rank-sum test was used to assess differences between the two groups. (B) RNF126, via MIDN, regulates EGR1-controlled signaling molecules, including p53 and PTEN. Immunoblotting was performed on lysates from wild-type TCam-2 cells, RNF126-knockout TCam-2 cells, and RNF126-knockout TCam-2 cells reconstituted with RNF126. (C) Protein levels of RNF126 and MIDN were assessed by immunoblotting in wild-type or MIDN-knockout TCam-2 cells with or without stable RNF126 expression. (D) RNF126 suppresses tumor growth in nude mice. TCam-2 cells of the indicated genotypes were inoculated subcutaneously, and the tumor volume was measured with a vernier caliper every 10 days. ** P < 0.01; ns, not significant; n = 6 mice per group. (E,F) Representative images of xenograft tumors harvested 50 days after the injection of TCam-2 cells of the indicated genotypes (E). The tumor weights were shown as the mean ± SD and evaluated by two-tailed unpaired t tests (F). **P < 0.01; ns, not significant; n = 6 mice per group. (G) RNF126, via MIDN, regulates EGR1-controlled signaling molecules, including p53 and PTEN, within xenograft tumors. The protein levels of the indicated proteins were detected by immunoblotting in xenograft tumors, as shown in (D). (H) Model depicting how the RNF126-MIDN ubiquitin-signaling axis regulates the EGR1 signaling pathway.

Pan-cancer analysis of UCSC Xena RNA-seq data revealed that in testicular germ-cell tumors (TGCTs), MIDN is highly expressed ( Figure 4A), whereas RNF126 is expressed at low levels ( Supplementary Figure S1A). EGR1, a transcription factor, is a direct target of MIDN-mediated degradation and functions as a pivotal downstream molecule within the MIDN regulatory network. By integrating EGR1 target gene annotations from the TRRUST, ENCODE, and FIMO databases, 51 high-confidence targets were defined, including the tumor-suppressor genes *TP53* and *PTEN* ( Supplementary Figure S1B). In TCam-2 cells, *RNF126* knockout increased MIDN abundance while reducing the protein levels of EGR1 and its downstream tumor suppressors p53 and PTEN, which were reversed upon re-introduction of wild-type RNF126 ( Figure 4B). The protein levels of RNF126 and MIDN were first verified in wild-type or *MIDN*-knockout TCam-2 cells, with or without stable RNF126 overexpression ( Figure 4C), and these lines were then used for xenograft assays. *In vivo*, RNF126 overexpression markedly retarded tumor growth; this suppressive effect was abolished when MIDN was concurrently ablated ( Figure 4D). At 50 days post-injection, tumors from the RNF126-OE group were significantly lighter than those from the control group were, whereas additional *MIDN* knockout abolished the effect of RNF126 on tumors. Notably, compared with MIDN-intact tumors, *MIDN* knockout alone resulted in significantly fewer tumors ( Figure 4E,F). Immunoblotting of excised tumors confirmed that RNF126-mediated tumor suppression coincided with MIDN degradation, elevated EGR1, and consequent stabilization of p53 and PTEN ( Figure 4 G).

Collectively, these data delineate a ubiquitin signaling cascade in which RNF126 tags MIDN for proteasomal degradation, thereby derepressing EGR1 and enabling the downstream accumulation of tumor-suppressor proteins such as p53 and PTEN ( Figure 4H).

## Discussion

Protein homeostasis is tightly regulated by the ubiquitin–proteasome system (UPS), which primarily targets lysine residues for ubiquitination and subsequent degradation [ 2 , 24– 30]. However, emerging evidence suggests that non-canonical ubiquitination of non-lysine residues, such as cysteine, serine, and threonine residues, also plays a role in regulating protein stability and function [ 14, 31]. Our study identified MIDN, a recently discovered regulator of ubiquitin-independent proteasomal degradation [16], as a substrate of the E3 ubiquitin ligase RNF126. Notably, this ubiquitination occurs predominantly on non-lysine residues (C230, C236, S237, T239, and S241), revealing a previously unrecognized layer of post-translational regulation.

RNF126-mediated ubiquitination of MIDN leads to its degradation via the 26S proteasome, as evidenced by the stabilization of MIDN in *RNF126*-knockout cells and the restoration of its turnover upon reintroduction of wild-type RNF126 ( Figure 3C). This regulatory axis is functionally significant, as it modulates the abundance of MIDN and thereby influences its downstream targets, particularly EGR1 and its downstream tumor suppressors p53 and PTEN ( Figure 4B). While MIDN has been reported to undergo ubiquitin-independent degradation via its Ubl domain 16], our findings indicate that, under the steady-state conditions used here, its stability is governed primarily by RNF126-mediated ubiquitination, a conclusion that is consistent with the recent demonstration that Midnolin ubiquitination is required for its proteasomal degradation [32]. This finding underscores that the relative contribution of ubiquitin-dependent versus ubiquitin-independent pathways is context-dependent and might vary with cell type, stress status, or substrate availability. Our *in vivo* xenograft studies further demonstrated that this axis has a tangible impact on tumor growth, particularly in testicular germ-cell tumors (TGCTs), where MIDN is frequently upregulated ( Figure 4D–F).

These findings expand the functional repertoire of RNF126, which has previously been implicated in the ubiquitination of multiple proteins, including membrane-associated proteins involved in endosomal trafficking [ 33– 35]. Physiologically, RNF126 is rapidly downregulated when normal epithelial cells detach from the extracellular matrix or when MAPK/ERK activity decreases [36]. Pathologically, however, the same pathway is chronically re-activated in cancer: oncogenic MEK-ERK signaling, E2F1 induction, promoter hypomethylation, and metabolic or oxidative stress all converge to keep RNF126 transcriptionally or post-translationally elevated [ 36– 38]. Our work identified RNF126 as a critical regulator of nuclear proteins involved in transcriptional control and tumor suppression. Moreover, the identification of non-lysine ubiquitination as a determinant of MIDN stability challenges the traditional view of ubiquitin signaling and suggests that similar mechanisms may govern other short-lived or regulatory proteins.

From a therapeutic perspective, the RNF126-MIDN-EGR1 axis is a promising target. Elevated MIDN expression in cancers, including TGCTs, may promote tumor progression by suppressing key tumor-suppressor pathways [ 19, 39, 40]. Strategies aimed at enhancing RNF126 activity or disrupting MIDN stability could restore EGR1-mediated tumor suppression, offering a novel avenue for cancer therapy. Additionally, the non-canonical ubiquitination sites identified in MIDN may serve as biomarkers for tumor subtypes or as targets for small-molecule interventions.

In conclusion, our study reveals a unique ubiquitin-mediated regulatory mechanism that governs MIDN stability and downstream signaling. By linking RNF126 to MIDN degradation and tumor suppression, we provide new insights into the complex interplay between ubiquitin signaling and proteostasis in cancer biology.

## Supporting information

25857Supplementary_Table_S2

25857supplementary_Data

## Supplementary Data

Supplementary data are available at *Acta Biochimica et Biophysica Sinica* online.
