# Supplementary material for: RNF126 writes a non-canonical ubiquitin code on midnolin to tune protein stability: RNF126 writes a non-canonical ubiquitin code on midnolin
Source: Acta Biochim Biophys Sin (Shanghai). 2026 Jan 7;58(5):1170–8. doi: 10.3724/abbs.2025232 (PMC13191471; doi:10.3724/abbs.2025232)
Supplement: 25857supplementary_Data [file 25857supplementary_Data.docx]

**Supplementary Table 1.** **Sequences of the sgRNA primers for RNF126 and MIDN**

| sgRNA primers | Sequence (5′→3′) |
| --- | --- |
| sgRNF126-F | CACCGCCTATGGACTACGCCTGGG |
| sgRNF126-R | AAACCCCAGGCGTAGTCCATAGGC |
| sgMIDN-F | CACCGAGGTCGTAGCGGGTGCCCG |
| sgMIDN-R | AAACCGGGCACCCGCTACGACCTC |


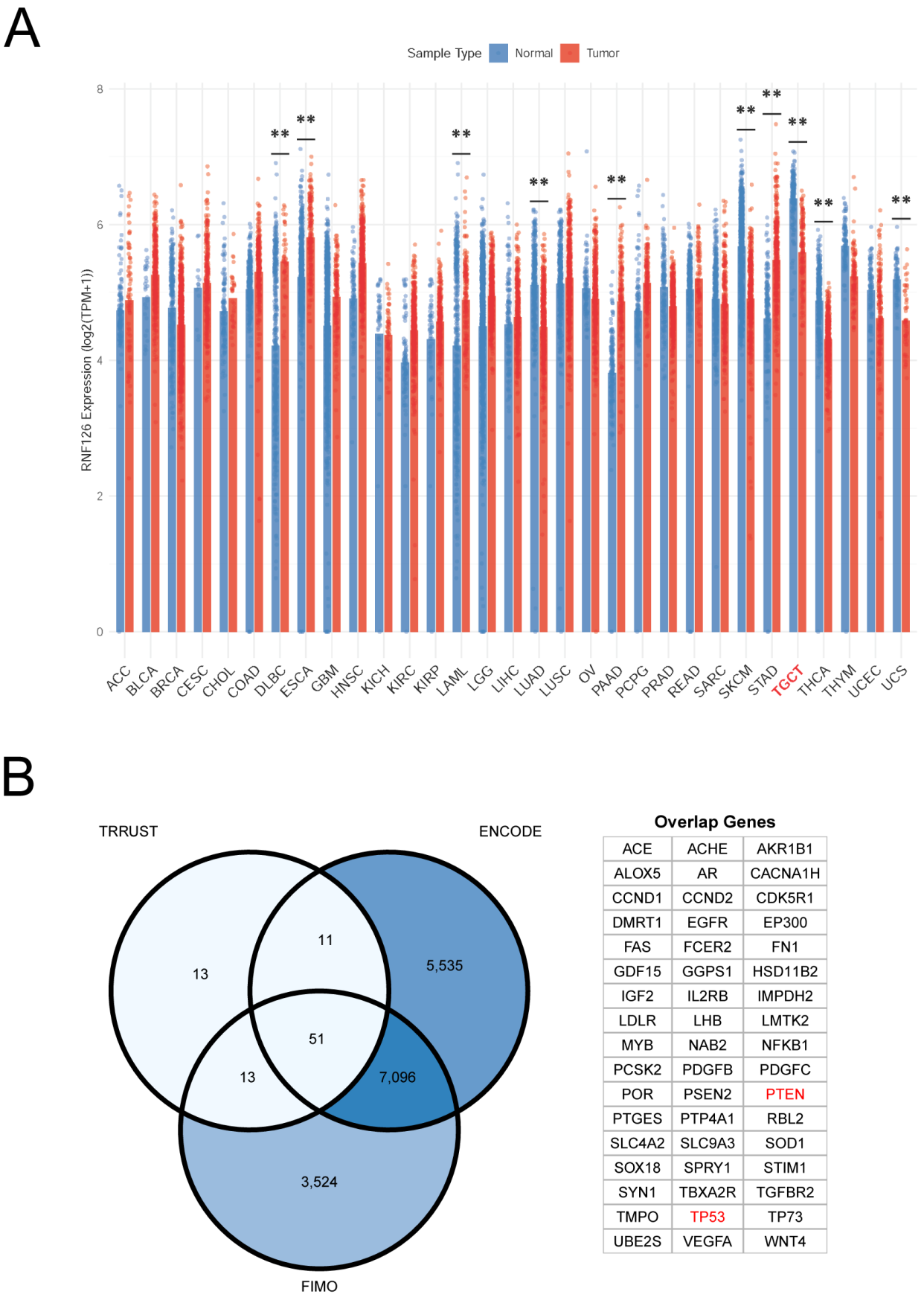


**Supplementary Figure S1. RNF126 regulates the downstream signaling pathways of MIDN-mediated, ubiquitin-independent degradation**  (A) Analysis of RNF126 expression across different tumor types. Download expression data containing TCGA and GTEx samples from the UCSC Xena database, with GTEx samples serving as supplementary normal controls for various cancer types. Use R's ggplot2 package to generate bar plots, and apply the Wilcoxon rank-sum test to assess differences between the two groups. (B) Uncovering EGR1 target genes via multi-database integration. Integrative in-silico prediction of EGR1 target genes by intersecting high-confidence sets derived from TRRUST (literature-curated), ENCODE (ChIP-seq-based), and FIMO (motif-scanning) databases, followed by Venn visualization of the tripartite overlap.
